# Supplementary material for: Inhaled nebulized glatiramer acetate against Gram-negative bacteria is not associated with adverse pulmonary reactions in healthy, young adult female pigs
Source: PLoS One. 2019 Oct 10;14(10):e0223647. doi: 10.1371/journal.pone.0223647 (PMC6786617; doi:10.1371/journal.pone.0223647)
Supplement: Attachment — Submitted filename: Response to Reviewers.pdf [file pone.0223647.s002.pdf]

Aarhus, August 27, 2019

Response to reviewers and editorial office

Journal requirements:

1. When submitting your revision, we need you to address these additional requirements.

Please ensure that your manuscript meets PLOS ONE's style requirements, including those for file naming. The PLOS ONE style templates can be found at http://www.journals.plos.org/plosone/s/file?id=wjVg/PLOSOne_formatting_sample_main_body.pdf and http://www.journals.plos.org/plosone/s/file?id=ba62/PLOSOne_formatting_sample_title_authors_affiliations.pdf

REPLY: Thank you for noticing this. We have changed "Figure" to "Fig" in the manuscript. We have addressed the template again, however if further changes are required please let us know.

2. Please include captions for your Supporting Information files at the end of your manuscript, and update any in-text citations to match accordingly. Please see our Supporting Information guidelines for more information: http://journals.plos.org/plosone/s/supporting-information.

REPLY: We have decided to apply to full data availability instead of a supplementary section. Thus, all data submitted along the manuscript can be found at figshare.com, DOI 10.6084/m9.figshare.9003164, at the time of publication (not available until then but corresponding to the files submitted).

3. Please note that all PLOS journals ask authors to adhere to our policies for sharing of data and materials: https://journals.plos.org/plosone/s/data-availability. According to PLOS ONE's Data Availability policy, we require that the minimal dataset underlying results reported in the submission must be made immediately and freely available at the time of publication. As such, please remove any instances of 'unpublished data' or 'data not shown' in your manuscript and replace these with either the relevant data (in the form of additional figures, tables or descriptive text, as appropriate), a citation to where the data can be found, or remove altogether any statements supported by data not presented in the manuscript."

REPLY: All data related to the manuscript will be publicly available here: DOI 10.6084/m9.figshare.9003164. In the manuscript, the phrase "*data not shown*" has been replaced with "*data available here: DOI 10.6084/m9.figshare.9003164*".

4. We note that you have a patent relating to material pertinent to this article. Please provide an amended statement of Competing Interests to declare this patent (with details including name and number), along with any other relevant declarations relating to employment, consultancy, patents, products in development or modified products etc. Please confirm that this does not alter your adherence to all PLOS ONE policies on sharing data and materials, as detailed online in our guide for authors http://journals.plos.org/plosone/s/competing-interests by including the following statement: "This does not alter our adherence to PLOS ONE policies on sharing data and materials." If there are restrictions on sharing of data and/or materials, please state these. Please note that we cannot proceed with consideration of your article until this information has been declared.

This information should be included in your cover letter; we will change the online submission form on your behalf.

Please know it is PLOS ONE policy for corresponding authors to declare, on behalf of all authors, all potential competing interests for the purposes of transparency. PLOS defines a competing interest as anything that interferes with, or could reasonably be perceived as interfering with, the full and objective presentation, peer review, editorial decision-making, or publication of research or non-research articles submitted to one of the journals. Competing interests can be financial or non-financial, professional, or personal. Competing interests can arise in relationship to an organization or another person. Please follow this link to our website for more details on competing interests: http://journals.plos.org/plosone/s/competing-interests

REPLY: We certainly intended to declare all necessary information and we adhere to all PLOS ONE's policies. We have now modified the statement as to the following:

> *"I have read the journal's policy and the authors of this manuscript have the following competing interests: HC, EP, and TVJ are the inventors on the US patent #10166253 "Positively charged co-polymers for use as antimicrobial agents" and an identically entitled application to the European Patent Office (EP3185882A1) describing the use of GA as an antimicrobial agent. The patent is owned by Aarhus University and Region Midtjylland, Aarhus, Denmark and licensed by Cycle Pharmaceuticals Ltd, Cambridge, UK. Neither the patent nor the mentioned application alters our adherence to all PLOS ONE policies on sharing data and materials. The other authors declare no competing interests."*

5. We note that you have included the phrase "data not shown" in your manuscript. Unfortunately, this does not meet our data sharing requirements. PLOS does not permit references to inaccessible data. We require that authors provide all relevant data within the paper, Supporting Information files, or in an acceptable, public repository. Please add a citation to support this phrase or upload the data that corresponds with these findings to a stable repository (such as Figshare or Dryad) and provide and URLs, DOIs, or accession numbers that may be used to access these data. Or, if the data are not a core part of the research being presented in your study, we ask that you remove the phrase that refers to these data.

REPLY: (As mentioned above) All data related to the manuscript will be publicly available at figshare.com at DOI: 10.6084/m9.figshare.9003164. In the manuscript, the phrase "*data not shown*" has been replaced with "*data available here: DOI 10.6084/m9.figshare.9003164*".

While revising your submission, please upload your figure files to the Preflight Analysis and Conversion Engine (PACE) digital diagnostic tool,https://pacev2.apexcovantage.com/. PACE helps ensure that figures meet PLOS requirements. To use PACE, you must first register as a user. Registration is free. Then, login and navigate to the UPLOAD tab, where you will find detailed instructions on how to use the tool. If you encounter any issues or have any questions when using PACE, please email us at figures@plos.org. Please note that Supporting Information files do not need this step.

REPLY: The figure files FIG1 and FIG2 has now been adjusted by PACE and uploaded along the manuscript resubmission.

Editor Comments: Please address all the comments of the reviewers

REPLY to editor: All comments have now been addressed.

Reviewer #1: The current study looked at the safety of glatiramer acetate (GA) intratracheal nebulization in healthy, young adult female pigs. While the experiments, statistics, and most analyses were performed to a high technical standard and described in sufficient detail, the reviewer has some concerns that may affect the conclusions presented. Additional comments are included in the revised manuscript.

1. In general safety studies includes both sexes. The current study only looked at female pigs. Why were males not included? Please explain and justify.

2. Barbiturates tend to relax smooth muscle during anesthesia. Could the use of propofol have influenced the measurement of Ppeak?

3. In a clinical setting, administration of GA would have been given by oro-nasal route; right? In the current study, this would have changed the assessment of safety parameters, including, at the very least, histopathological evaluation of the upper respiratory tract. Why was the intratracheal route chosen over the oro-nasal route? Please explain and justify.

4. Why not do flow cytometry in lung tissue and look for the various cell types (monocytes, T-cells, and others), including eosinophils and plasma cells, which play an important role in the generation of an immediate and delayed reaction that can lead to broncho- and bronchiolo-constriction? See reference: Yu Y-RA, O'Koren EG, Hotten DF, Kan MJ, Kopin D, Nelson ER, et al. (2016) A Protocol for the Comprehensive Flow Cytometric Analysis of Immune Cells in Normal and Inflamed Murine Non-Lymphoid Tissues. PLoS ONE 11(3): e0150606. doi:10.1371/ journal.pone.0150606

5. Who served as the study pathologist for the study?
a. What were his/hers credentials?
i. European College of Veterinary Pathology?
ii. American College of Veterinary Pathology?
b. Was the study read blindly (masked) at least initially?
c. Were there any fresh samples collected for analysis? If so, what analysis, if any?
i. This would have been a good opportunity to do microbial isolation, flow cytometry, PCR, etc.
d. Was the lungs infused with fixative?
e. What did the histopathological evaluation consisted of? What were the parameters observed? Their incidence? Their severity? Please be specific.

Example of how I would have approached this:
i. "Microscopic exam consisted of evaluation of the trachea, bronchi, bronchioles, respiratory bronchioles, and alveolar sacs from all lung lobes (the pig has 7 lobes) for the presence or absence of significant tissue alterations (lesions), including but not limited to inflammation, degeneration, and necrosis. Microscopically, lesion (tissue alteration) incidence, severity, and distribution were recorded. Histopathologic severity scores were assigned as grades 0 (no significant histopathological alterations); 1 (minimal); 2 (mild); 3 (moderate); or 4 (severe) based on an increasing extent and/or complexity of change, unless otherwise specified. Lesion distribution was recorded as focal, multifocal, or diffuse, with distribution scores of 1, 2, or 3, respectively."

In addition, the histopathology data needs to be presented in a tabular format.

Example:

ii. “The incidence, severity, and distribution of significant microscopic findings are summarized on the Project Tabulated Animal Data - Summary - Microscopic Findings, in which the numbers of animals per group and lesions per group are indicated. Project Tabulated Animal Data – Individual Animals – Microscopic Findings present the microscopic findings for individual animals by group. The codes and abbreviations used as entries in these tables are explained at the bottom of each table.”
iii. More information can be found at: Kenneth A. Schafer, John Eighmy, James D. Fikes, Wendy G. Halpern, Renee R. Hukkanen, Gerald G. Long, Emily K. Meseck, Daniel J. Patrick, Michael S. Thibodeau, Charles E. Wood, Sabine Francke. 2018. Use of Severity Grades to Characterize Histopathologic Changes. Toxicol Pathol 46, 256-265; https://doi.org/10.1177/0192623318761348

6. Did you do histopathology at the injection site? If not, then why not? Subcutaneous lipoatrophy, necrosis, and others have been associated with administration of GA.

7. Data from pig #18 had a wide range in Ppeak. Although this may not be clinically significant, this was a healthy pig nevertheless. I would expect the difference be more accentuated in sick animal. Is this interpretation correct?

8. Pigs coming directly from the farm, such as the ones from the current study, usually have some sort of bronchial-associated lymphoid hyperplasia, often secondary to Mycoplasma ssp. infections, which potentially makes the airways hypersensitive to stimuli, such as GA. Did you see any of that? Other potential lung infections from the farm include Bordetella, Haemophilus, Streptococcus, several viruses, and parasites. Were these pigs checked for non-clinical infections? Again, who did the evaluation of the lungs? What were his/hers credentials?

REPLY to Reviewer #1:
Thank you for taking the time to address the potential issues and help us improve the manuscript. We are very grateful for your thorough assessment and good questions that were raised.

1. We recognize that a general safety study would optimally include both sexes. However, boars can be more aggressive and require individual stalls that are four times bigger according to the regulations. This causes some practical challenges as the animals must not be isolated (cf. the Danish law in accordance with the Directive 2010/63/EU, European Parliament on the protection of animals used for scientific purposes) and is associated with high costs. Thus, we chose not to include males in this preliminary study. To underline this, we accept your suggestion to change the title to the following:

> *“**Inhaled nebulized glatiramer acetate against Gram-negative bacteria is not associated with adverse pulmonary reactions in healthy, young adult female pigs**”*

And we inserted the following in the discussion line 302-304 and 304-05:

> *“We acknowledge that including only females is a limitation; however, boars were excluded in this preliminary study as they are more aggressive and require bigger, individual stalls according to the regulations [11,12].”*
>
> *”… within the modest number of animals included limited to females …”*

2. Correctly, barbiturates can relax smooth muscle and therefore cause bronchodilation, also in healthy individuals. Therefore, the study protocol is designed to avoid the known bronchoconstrictive effect of intubation as well as the bronchodilating effect of propofol. As such, the intervention (inhalation or s.c. injection) is initiated at least 30 minutes after induction of anaesthesia and intubation, at a time point where the pigs are fully stable regarding hemodynamics and ventilation. Thus, we expect that any changes in these parameters at the time of intervention will be caused by the intervention. We inserted the following in the manuscript methods section line 118-20:

> *"Following intubation and anaesthesia induction, the animals where observed for at least 30 minutes prior to the intervention, which was initiated at a time point where they were fully stable regarding haemodynamics and ventilation."*

3. The nebulizing inhalation therapy technique we use for, e.g., CF patients is mainly oral, not oro-nasal, e.g., the ultrasound eFlow rapid nebulizer system. We agree with the reviewer that the optimal administration route would be oral or oro-nasal on awake animals, however, to our knowledge no such devices exist for pigs. If so, they would have to be awake and would not likely cooperate. Furthermore, our primary measure was Ppeak recorded from the ventilator requiring general anaesthesia. Thus, we chose the intratracheal route, and our question if GA can be inhaled without inducing local inflammation was therefore limited to the main bronchi including carina and peripheral lung tissue. The intratracheal route would introduce a bias towards a higher concentration administered to the lungs compared to the oral route, if any. The following has been inserted in the methods section "GA administration", line 129-31:

> *"The intratracheal route was chosen as the animals had to be in general anaesthesia connected to a ventilator during recording of pulmonary parameters."*

The following changes has been made in the discussion section:

Line 295-96: *"… neither where there any local infiltrate of inflammatory cells detected by lower respiratory tract histopathology*".

Line 339: "*airways*" has now been replaced with "*lower respiratory tract*".

4. Thank you for the reference and for bringing up this idea. We do not have experience with flow cytometry on tissue samples but will consider including it in our future work.

5. The study pathologist was Ida E. Holm, DMSc, consultant pathologist, head of department and associate professor at the Department of Pathology, Randers Regional Hospital, Randers, Denmark. In Denmark, it is common that human pathologists assist veterinarians regarding pathology. Ida E. Holm has agreed to be assigned as co-author.

Regarding question 5b-e: Yes, the tissue samples for histopathology were blinded when initially harvested, which is now mentioned in the methods section. The lungs were not infused with fixative, but the tissue samples were cut out in small sections immediately after euthanizing. No fresh samples were taken for microbiological evaluation, as this was not the study purpose. Limited to the parameters investigated here (clinical- and vital parameters, lung parameters and histopathology) we did not detect any signs of subclinical infections, but we acknowledge the value of addressing this more specifically in our future studies (this matter will be more thoroughly discussed under question #8).

The histopathology exam evaluated if there were any significant histopathological alterations, including but not limited to inflammatory infiltrate, as done routinely by our clinical pathology department. We expected this to be sufficient, as the histopathology was only supplementary to our main focus Ppeak. As the histopathology is thoroughly addressed by the reviewer, we have had a discussion with our pathologist, who underlines that the study samples were completely normal. Thus, no additional analyses or classifications were done. However, we will provide images of the normal mucosa samples if this is required. Also, considering the mucosa area onto which the GA is administered by inhalation, we expect the local concentrations to be lower compared to subcutaneous administration, where the concentrations are high immediately after injection. By inhalation, the drug is spread out onto the mucosa, why we did not expect an inflammatory response with this administration form even with the high dosage administered (cf. Discussion, line 272-80).

However, we appreciate the good suggestions on how to more thoroughly address the histopathology when assessing research questions and will adhere to this in our future work. As requested, we have summarized the results in a tabular form, which can be found at figshare.com, DOI 10.6084/m9.figshare.9003164, at the time of publication:

	**Pig #1**	**Pig #2**	**Pig #3**	**Pig #4**	**Pig #5**	**Pig #6**	**Pig #7**	**Pig #8**
**Intervention:**	Mannitol	Mannitol	GA s.c.	GA s.c.	GA inh.	GA inh.	GA inh.	GA inh.
Histopathology:								
Carina, main bronchi:	0	0	0	0	0	0	0	0
Peripheral lung tissue:	0	0	0	0	0	0	0	0

Table R1. Histopathologic severity scores were assigned as grades 0 (no significant histopathological alterations); 1 (minimal); 2 (mild); 3 (moderate); or 4 (severe).

In the manuscript, we have added the following (Methods, line 177-79):

> *"Microscopic examination included evaluation of the main bronchi (carina) and peripheral lung tissue for the presence or absence of significant tissue alterations and signs of inflammatory infiltrate."*

6. Indeed, local inflammation is a well-known side effect to subcutaneous GA administration as mentioned in the results section line 198. However, it was not our purpose to investigate this why we did not do histopathology at the injection site. GA has been administered subcutaneously for decades and our focus here was pulmonary and systemic effects.

7. Thank you for pinpointing this. We reckon you are referring to the raw data from pig #8? If so, the slightly wider range results from a single outlier prior to the intervention on one experimental day (cf. Pre1, 26/May in the file "GA pigs raw data.xlsx"). However, please also refer to the figures below not included in the submission but available from Figshare.com at the time of publication. As highlighted in Table R1 below, the pre-intervention Ppeak level is generally low for this animal.

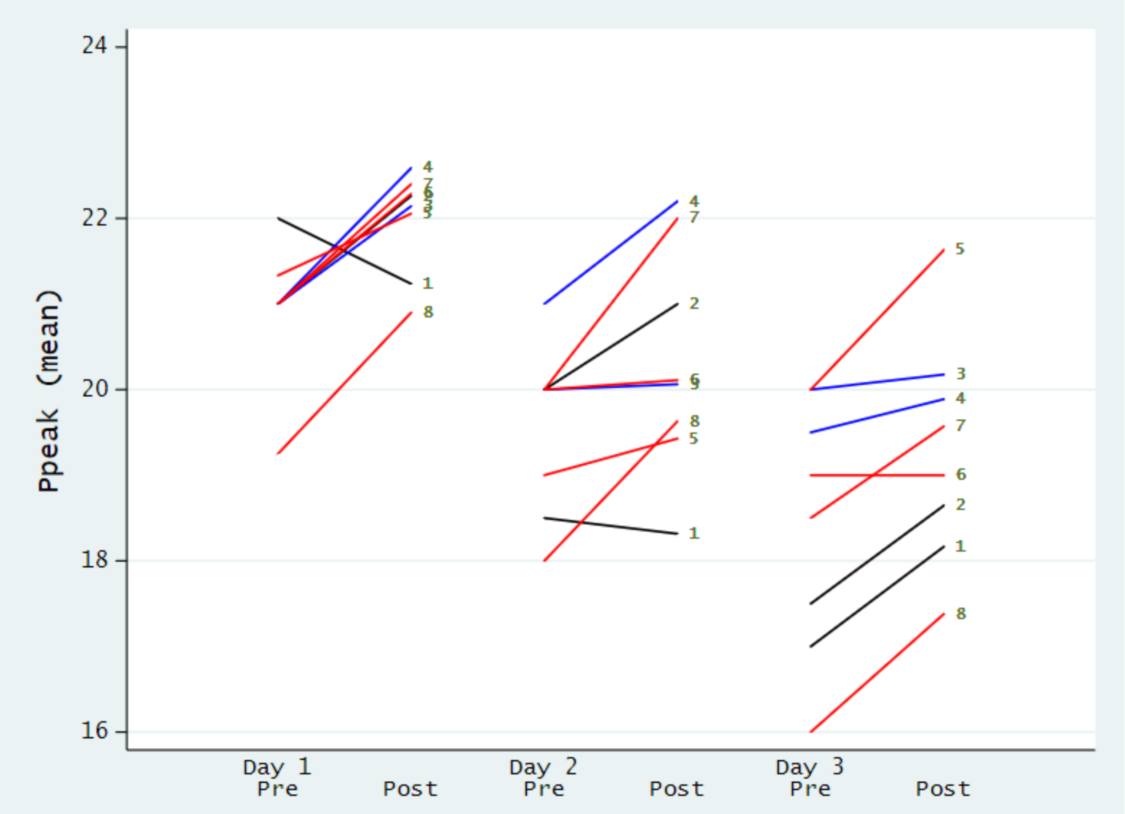

Fig. R1. Pre- and post-intervention Ppeak (mean) for pig # 1-8 on Day 1, 2 and 3. Black: mannitol; blue: GA s.c.; red: GA inh. Note that the monitoring accuracy is ±2 cmH_2O, and that changes within this range are equivalent and clinically insignificant.

		n	mean	sd
Pig 1	Control 1, Day, 1, Pre	2	22.000	0.000
	Control 1, Day, 1, Post	17	21.235	0.437
	Control 1, Day, 2, Pre	2	18.500	0.707
	Control 1, Day, 2, Post	19	18.316	0.478
	Control 1, Day, 3, Pre	2	17.000	1.414
	Control 1, Day, 3, Post	18	18.167	0.383
Pig 2	Control 1, Day, 1, Pre	1	21.000	
	Control 1, Day, 1, Post	19	22.263	0.452
	Control 1, Day, 2, Pre	2	20.000	0.000
	Control 1, Day, 2, Post	19	21.000	0.333
	Control 1, Day, 3, Pre	2	17.500	0.707
	Control 1, Day, 3, Post	17	18.647	0.493
Pig 3	Control 2, Day, 1, Pre	1	21.000	
	Control 2, Day, 1, Post	14	22.143	1.027
	Control 2, Day, 2, Pre	1	20.000	
	Control 2, Day, 2, Post	16	20.063	0.443
	Control 2, Day, 3, Pre	2	20.000	0.000
	Control 2, Day, 3, Post	17	20.176	0.393
Pig 4	Control 2, Day, 1, Pre	2	21.000	0.000
	Control 2, Day, 1, Post	17	22.588	0.870
	Control 2, Day, 2, Pre	1	21.000	
	Control 2, Day, 2, Post	15	22.200	1.207
	Control 2, Day, 3, Pre	2	19.500	0.707
	Control 2, Day, 3, Post	18	19.889	0.676
Pig 5	Intervention, Day, 1, Pre	3	21.333	0.577
	Intervention, Day, 1, Post	18	22.056	0.802
	Intervention, Day, 2, Pre	2	19.000	0.000
	Intervention, Day, 2, Post	21	19.429	0.746
	Intervention, Day, 3, Pre	4	20.000	0.000
	Intervention, Day, 3, Post	19	21.632	1.300
Pig 6	Intervention, Day, 1, Pre	3	21.000	0.000
	Intervention, Day, 1, Post	21	22.286	0.717
	Intervention, Day, 2, Pre	1	20.000	
	Intervention, Day, 2, Post	18	20.111	0.323
	Intervention, Day, 3, Pre	2	19.000	0.000
	Intervention, Day, 3, Post	21	19.000	0.000
Pig 7	Intervention, Day, 1, Pre	7	21.000	0.000
	Intervention, Day, 1, Post	20	22.400	0.503
	Intervention, Day, 2, Pre	1	20.000	
	Intervention, Day, 2, Post	20	22.000	1.376
	Intervention, Day, 3, Pre	2	18.500	0.707
	Intervention, Day, 3, Post	21	19.571	1.076
Pig 8	Intervention, Day, 1, Pre	4	19.250	0.500
	Intervention, Day, 1, Post	20	20.900	0.968
	Intervention, Day, 2, Pre	2	18.000	0.000
	Intervention, Day, 2, Post	19	19.632	0.597
	Intervention, Day, 3, Pre	4	16.000	2.160
	Intervention, Day, 3, Post	21	17.381	0.590

Table R2. Number (n), mean and standard deviation (sd) of each animal pre- and post-intervention. Control 1: mannitol; Control 2: GA s.c.; Intervention: GA inh.

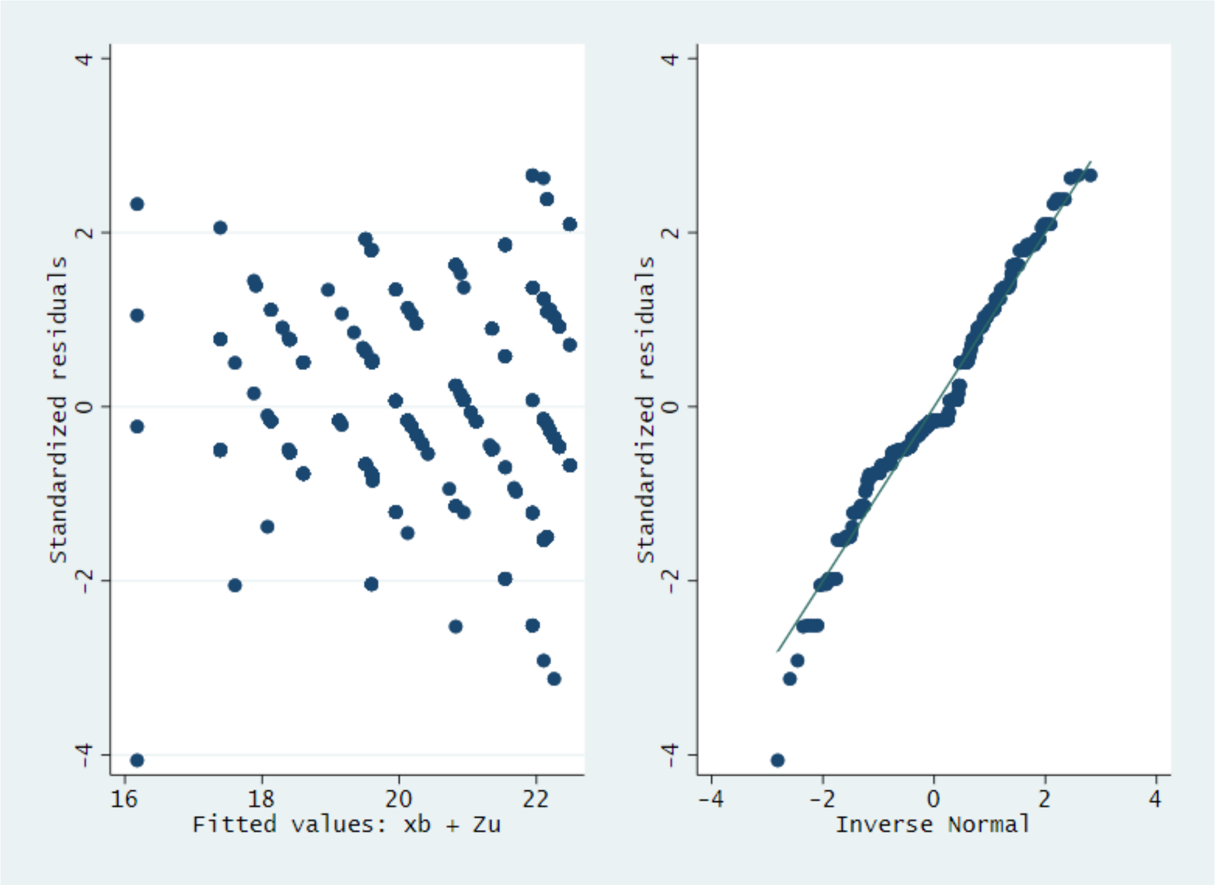

Fig. R2: Left: Standardized residuals against fitted values. Right: QQ-plot of standardized residuals normally distributed except for one outlier identified as "Intervention, Pig 8, Day 3, Pre").

8. Indeed, it is a recognized problem that study animals can have subclinical infections potentially influencing results. However, the swine herds of Denmark are renowned to be of high-quality regarding health control and biosecurity (internal and external infection control). Danish pig production complies to strict rules ensuring that the herds are free of a variety of pig diseases. 78 % of all pigs born in Denmark and 100 % of breeding stocks has Specific-Pathogen-Free (SPF) status, meaning that they are verified free of numerous diseases, including common lung infections caused by *Mycoplasma hyopneumoniae*, *Actinobaccillus pleuropneumoniae spp.*, PRRS-viruses etc. (more information regarding the Danish Health Status Management can be found at http://spfsus.dk/en).

Additionally, our animals gained weigh throughout the study period also indicating that they were generally in good health. We did not take any additional samples to check our pigs for colonization specifically, however as mentioned previously, there were no signs of respiratory symptoms, altered vital parameters including temperature, hypersensitivity nor inflammation in the histopathology limited to the sections investigated. Thus, we have no reason to suspect that any potential colonization altered our results. However, we are aware that this matter could be specifically addressed in our future investigations, where it is relevant in the context of a potential effect of GA inhalation on pulmonary infections.

Reviewer #2: The manuscript “Inhaled nebulized glatiramer acetate against Gram-negative bacteria does not induce pulmonary side effects in adult pigs” by Skovdal et al describes results of assessing the side effects on respiratory exposure of animals (pigs) with therapeutically ascertained doses of the random co-polymer glatiramer acetate (GA). The compound is currently in use as an FDA approved compound to treat MS patients by intravenous injections though observed to have anti-inflammatory and leukocytic activities. GA was subsequently see to possess antimicrobial activity similar to the cationic peptide LL-37 The manuscripts starts with the premise that the drug is inherently safe within the body.

Major Comments

I feel, in its present form, the narrow focus of the preliminary work, investigating the safety in the putative use of GA as a therapeutic may be more appropriate in journals focused on such studies. As such the quantum of work currently presented in the manuscript does not reach the threshold of an independent publication and would be more suited as preliminary work/data accompanying a broader investigation, namely the therapeutic benefit of GA in animal models of respiratory infection.

Therefore, this reviewer feels that in the current form the manuscript cannot be published and requires a major revision.

Minor comments on manuscript

1. There are few technical deficiencies in the manuscript. Though the control groups of animals used (n=2) are small in number and may be straining statistical results.
2. The language is sufficiently clear for the most part except for some clarity required in animal number assignments in the Materials (line 95: 12 pigs; Line 101 “four pigs used for pilot studies” Line “8 pigs divided into 3 groups”)
3. The Results section begins without any compiled description of the experimental format (the descriptions of the experiments are described in bits and pieces among various parts of Materials section).
4. The manuscript may be better served if the background of the earlier studies (some conducted by the authors group) is elaborated in the introduction to increase the contextual significance of the manuscript.

REPLY to Reviewer #2:

Thank you for taking the time to address potential issues and to help us improve the manuscript.

Major comments reply: We have submitted the work to PLOS ONE Antimicrobial Resistance Collection because we think that the questions addressed extend beyond the specific antimicrobial peptide studied here and is of interest to the readers of PLOS ONE. We agree with the reviewer that the next step will be to investigate the therapeutic benefits of GA in an animal model of respiratory infection. However, our focus here was to address the pulmonary safety of GA inhalation, and to suggest this administration route of GA to be further studied for different clinical applications. We consider the questions addressed in the present work fundamental and relevant for inhalation of antimicrobial peptides in general.

Minor comments reply:

1. Thank you for acknowledging this. Regarding the number of animals in control groups, we did not have the opportunity to include more animals at that time. We believe that we are very clear in our conclusion regarding this, underlining that this is a preliminary study (Discussion, line 304-05):

> *“… within the modest number of animals included…”.*

2. Thank you for pinpointing this, the phrase has now been changed to (line 102-04):

"Four animals were used for pilot studies and excluded from the final results. The remaining eight pigs were divided into three groups: …"

3. The experimental methods used is now briefly mentioned in the result sections:

Line 202: *"Measured by a transcutaneous probe the pO_2 remained stable in all animals …"*

Line 204-05: *"The primary measure of interest was P_{peak} recorded by the ventilator …"*

Line 235: *"Routine photometry of blood samples showed that all …"*

Line 246-47: *"As GA has an immunomodulating effect when administered s.c., we studied if inhaled GA influenced white blood cell subsets. This was done by immune cell analyses by flow cytometry."*

4. Regarding earlier studies highly important to the manuscript significance, we believe that we have already elaborated on this in the introduction line 68-74:

"Recently, GA was discovered to have chemical and biophysical properties in common with the naturally occurring antimicrobial peptide LL-37, notably the ability to disrupt biological membranes [9]. The close resemblance between the two compounds hypothesized that the action of GA was due to interaction with cell membranes and that bacteria could be susceptible to GA. We confirmed that GA indeed disrupted bacterial cell membranes, particularly the Gram-negative Pseudomonas aeruginosa, Escherichia coli and Acinetobacter baumannii and to a lesser extent the Gram-positive Staphylococcus aureus [4]. …"

We deliberately kept this section brief, as our intention with this manuscript is to keep the main focus on the safety of inhaling GA, prior to further investigations on effect of GA inhalation on pulmonary infections. However, there is already rising evidence that GA increase the efficacy of other antibiotics against *Pseudomonas aeruginosa* (DOI: https://doi.org/10.1016/S1569-1993(19)30419-9), why we suggest future investigations on this purpose of GA in the discussion.

We thank all reviewers for their time and thorough work with our manuscript. We are grateful for all the excellent suggestions and comments, and for this opportunity to improve our manuscript and future research.

Sincerely,
Thomas Vorup-Jensen
